# Dynamin 1 Is Required for Memory Formation

**DOI:** 10.1371/journal.pone.0091954

**Published:** 2014-03-18

**Authors:** Mauro Fà, Agnieszka Staniszewski, Faisal Saeed, Yitshak I. Francis, Ottavio Arancio

**Affiliations:** Taub Institute for Research on Alzheimer's Disease and the Aging Brain, Columbia University, New York, New York, United States of America; University of Edinburgh, United Kingdom

## Abstract

Dynamin 1–3 isoforms are known to be involved in endocytotic processes occurring during synaptic transmission. No data has directly linked dynamins yet with normal animal behavior. Here we show that dynamin pharmacologic inhibition markedly impairs hippocampal-dependent associative memory. Memory loss was associated with changes in synaptic function occurring during repetitive stimulation that is thought to be linked with memory induction. Synaptic fatigue was accentuated by dynamin inhibition. Moreover, dynamin inhibition markedly reduced long-term potentiation, post-tetanic potentiation, and neurotransmitter released during repetitive stimulation. Most importantly, the effect of dynamin inhibition onto memory and synaptic plasticity was due to a specific involvement of the dynamin 1 isoform, as demonstrated through a genetic approach with siRNA against this isoform to temporally block it. Taken together, these findings identify dynamin 1 as a key protein for modulation of memory and release evoked by repetitive activity.

## Introduction

The role of dynamin in modulating synaptic activity has been widely debated since its discovery [1]. Dynamin is key in controlling various endocytotic pathways during neurotransmission [2], including the classical clathrin-mediated pathway [3]. Studies on dynamin and exocytosis have shown a block of exocytosis in the dynamin-impaired *shibire* Drosophila model [3]. Recent studies have demonstrated that inhibition of dynamin activity impairs evoked exocytosis occurring during low frequency stimulation without affecting spontaneous exocytosis in the mammalian brain [4]–[8]. Despite the intense research activity around the role of dynamin in synaptic transmission, it is not yet clear whether this involvement translates to normal animal behavior. Here we have investigated the effect of both pharmacologic and genetic block of dynamin function on memory formation. Furthermore, we have explored how blocking dynamin activity can affect neurotransmitter release evoked through repetitive activity that is known to underlie forms of synaptic plasticity that are, in turn, likely to be linked with memory formation.

## Materials and Methods

### Ethics Statement

All animals (C57BL/6J mice) were used and handled in strict accordance with good animal practice as defined by the Ethical Guidelines for Treatment of Laboratory Animals of Columbia University and specifically approved by Columbia University IACUC (protocol #AC-AAAB8674). Specific Pathogen Free, male mice, aged 4–5 months, were obtained from Jackson Laboratories. Mice were housed under a 12-h (8.00–20.00) light/dark cycle in a climate-controlled room (23°C±1) with *ad libitum* access to food and water. All efforts were made to minimize the number of animals used and their suffering. Animals were housed 4–5 per cage after weaning.

### Drugs and treatments

Dynasore, kindly provided by Tomas Kirchhausen (Harvard Medical School, Boston, USA) was dissolved in anhydrous DMSO to obtain a 200 mM stock concentration and then stored at −80°C. Working solutions were obtained by diluting, in dim light environment, aliquots of stock solutions in artificial cerebro-spinal fluid (ACSF) containing NaCl 124 mM, KCl 4.4 mM, Na_2_HPO_4_ 1 mM, NaHCO_3_ 25 mM, Glucose 10 mM, CaCl_2_ 2 mM, MgCl_2_ 2 mM, supplemented with 0.3% DMSO. Drug solution was then either perfused for 20 minutes onto hippocampal slice preparations or injected into dorsal hippocampi.

siRNA duplexes with a 5′ thiol on the sense strand were synthesized (Thermo Scientific Dharmacon, CO). The siRNA sequence against Mus musculus dynamin 1 was: “5′(S-S) UAA GUG UCA AUC UGG UCU C dTdT 3′” while the siRNA sequence for the control siRNA was “5′(S-S) CGU ACG CGG AAU ACU UCG AUU dTdT 3′”. Annealed siRNA duplexes were re-suspended in K^+^ buffer, incubated at 90°C and then treated with an equimolar mixture of TCEP (#20490, Thermo Scientific, USA), a strong reducing agent, at 20°C for 15 min. An equimolar ratio of monomeric Penetratin1 (#11PENA0500, MPI Biomedicals, USA) was then incubated at 65°C for 15 min with the siRNA mixture, followed by a further incubation at 37°C for 1 hr, as previously described [9]. 5′-siRNA/Penetratin1 linking was assessed by Tris-Borate PAGE, visualized with SyBrGold (#S-11494, Life Technologies). The efficacy of the siRNA to block endogenous dynamin 1 was previously validated on primary hippocampal neuronal cultures, prepared as previously described [9]. Dynamin detection was assessed by harvesting cell cultures upon extensive washing with fresh medium and then with cold Hank's Balanced Salt Solution. Tissue lysis was carried out in cold 3% lithium dodecylsulfate (#L-2274, Sigma Aldrich, USA) in 62.6 mM Tris buffer, pH 6.8, supplemented with 3X protease and phosphatase inhibitors (#05 892 970 001 cOmplete ULTRA Mini, and #04906837001 PhosStop, Roche Applied Science, IN, USA). Protein concentration was determined using the detergent-resistant protein concentration assay (BioRad DC kit, #500-0006). Equal amounts of protein (5 μg) were loaded on each lane of the 7% Tris-Acetate gel (Life Technologies) and then electrotransferred onto a nitrocellulose membrane (ProTran #BA79 0.1 μm, Whatman GmbH, Germany). Dynamin 1 was immunoblotted by using a rabbit polyclonal anti-dynamin1 antibody (1∶1000, #PA1-660, Affinity Biosciences) then probed with a horseradish peroxidase-conjugated donkey anti-rabbit secondary antibody (1∶20,000, JacksonImmuno Laboratories, USA). Dynamin signal detection was achieved using SuperSignal West Pico chemiluminescent substrate (#34080 Thermo Scientific Pierce, IL, USA) and band intensities were normalized to that of β-III-tubulin signal (1∶2000, #G7121, Promega, WI, USA), used as housekeeping protein for neuronal samples.

### Infusion technique

Bilateral cannulas were fixed to the skull, as previously described [10]. Briefly, six-to-eight days before behavioral testing, mice were implanted with intracerebral cannula for the intrahippocampal drug delivery. Pre-emptive analgesia was induced by carprofen and marcaine injection at the time of anesthesia induction. Local anesthesia at the implantation site was obtained by subcutaneous infiltration of marcaine at 2 mg/Kg post op. Analgesia was achieved through carprofen 5 mg/kg subcutaneous injection every 24 hours for 48 hours. When general anesthesia was obtained (Avertin 250 mg/kg i.p.), animals were placed in the stereotaxic frame. A 26-gauge guide cannula was lowered above the dorsal hippocampi (stereotaxic coordinates: AP = 2.4 mm, ML = 1.5 mm to a depth of 1.3 mm), and secured to the skull by acrylic dental cement (Paladur). After 6–8 days, mice were bilaterally injected (1.5 μl/injection over 1 minute) through intracerebral cannulas connected to a microsyringe by polyethylene tubing. To ensure sterility of the infused substances, drugs were diluted in filtered artificial cerebrospinal fluid. The injected substances were either with vehicle (ACSF +0.3% DMSO), or dynasore (80 μM, in vehicle medium), or siRNA (80 nM, in vehicle medium). For the experiments employing dynasore, mice received a single injection 20 minutes prior to the electric shock. For the siRNA experiments, however, mice were treated with either vehicle or siRNA against dynamin 1 or control siRNA against luciferase two times/day for three days; last administration was 12 hours before training. Mice were handled once a day for 3 days before behavioral experiments. During infusion, animals were handled gently to minimize stress. After infusion, the needle was left in place for 1 minute to allow diffusion. Histological localization of infusion cannulas was performed after behavioral testing, using a solution of 4% methylene blue.

### Behavioral Studies

All behavioral studies were performed by an observer who did not know the treatment group of the mice until the entire test had been completed, and conducted during the light phase (between 13:30–18:00) of the light/dark cycle (lights on 8:00–20:00).

Contextual and cued learning were assessed using the fear conditioning test, as previously described [10]. Briefly, mice were placed in a novel context (fear conditioning box) and exposed to a mild foot electric shock (2 s, 0.45 mA) together with a tone (30 s, 85 dB sound at 2800 Hz). The electric shock was delivered during the last two seconds of the auditory tone. Freezing was scored by using FreezeView software (SD Instruments, USA.) Learning was assessed 24 hours later by measuring freezing behavior for 5 minutes in the chamber in which the mice were trained in response to representation of the context (chamber) without auditory cue. Cued fear responses were assessed 48 hours after the shock by exposing the mice to the tone in a novel environment. Baseline behavior was monitored during the training phase. In separate experiments, sensory perception of the shock was determined through threshold assessment, as previously described [10]. The threshold to flinching (first visible response to shock), jumping (first extreme motor response), and screaming (first vocalized distress) was quantified for each animal by averaging of the shock intensity at which each animal manifested a behavioral response of that type to the foot shock (0.1 mA for 1 s). Shock intensity was increased at 30 s interval by 0.1 mA to 0.7 mA. Experiments were repeated in three different cohorts, grouped and analyzed together, in which controls and dynasore or siRNA treated mice were equally distributed.

### Slice Preparation and Electrophysiology

Mice were sacrified by cervical dislocation followed by decapitation. Transverse hippocampal slices (400 μm) were obtained by using a tissue-chopper and maintained in an interface chamber at 29°C. CA_3_–CA_1_ field excitatory post-synaptic potentials (*f*EPSP) were recorded by placing the stimulating electrode on CA_3_ fibers projecting to CA_1_ *stratum radiatum* where the recording electrode was placed [10]. Basal synaptic transmission was assayed by plotting the stimulus voltages (V) against the slope of *f*EPSPs to generate input-output relations before each experiment. For the long-term potentiation (LTP) experiments, a 30-minute baseline was recorded every minute at an intensity that evokes a response approximately 35–40% of the maximum evoked response. In most of the experiments LTP was induced using a theta-burst stimulation (4 pulses at 100 Hz, with the bursts repeated at 5 Hz and each tetanus including 3 ten-burst trains separated by 15 seconds). In a few experiments, LTP was induced through a different protocol (three tetani at 50 Hz, 1 sec each, 20 second interval between tetani) that is known to solely involve post-synaptic mechanisms [11]. Post-Tetanic Potentiation (PTP) was induced through the same theta burst tetanus used to induce LTP. Within the same slice, PTP was first recorded in absence of dynasore, then in its presence, and finally after its washout. In Synaptic Fatigue (SF) experiments, the slope of the evoked responses during a single 100 Hz-1 second stimulus train was measured every 10 stimuli and compared with the one associated with the first stimulus of the train. Similar to PTP, SF was first recorded in absence of dynasore, then in its presence, and finally after its washout within the same slice. SF experiments were also carried out in presence of GABA_A_ (picrotoxin, 30 μM) and GABA_B_ (SCH50911, 100 μM) receptor blockers, or in the presence of an AMPA receptor desensitization blocker (cyclothiazide, 100 μM). For both PTP measurement and SF assessment, the tetanic stimulation was applied in the constant presence of 100 μM D-APV, a competitive NMDA receptor antagonist. PPF analysis was carried out by providing a double stimulation delivered at different interstimulus intervals (10→1000 ms) and then comparing the ratio between the slope of the evoked response after the second stimulus (S**_2_**) vs. the slope of the evoked response obtained for the first stimulus (S**_1_**). Tetanic efficiency as an evaluation of the facilitation induced throughout the tetanus (an indirect index of neurotransmitter release) was evaluated through intra-burst analysis [12]. Briefly, the area of successive burst responses (charge) was expressed as a percentage fraction of the area of the first burst response. In these measurements each burst consisted of 4 pulses at 100 Hz with bursts repeated at 5 Hz. This measure normalizes differences between slices in the area of the first burst, thereby allowing for the detection of any effect that develops during the course of the stimulation train.

### Statistical analysis

All data are shown as mean ± SEM. Statistical tests included two-way ANOVA with repeated measures for multiple comparisons or two-tails Mann-Whitney test when appropriate.

## Results

### Dynamin is required for memory formation

**Figure 1:**
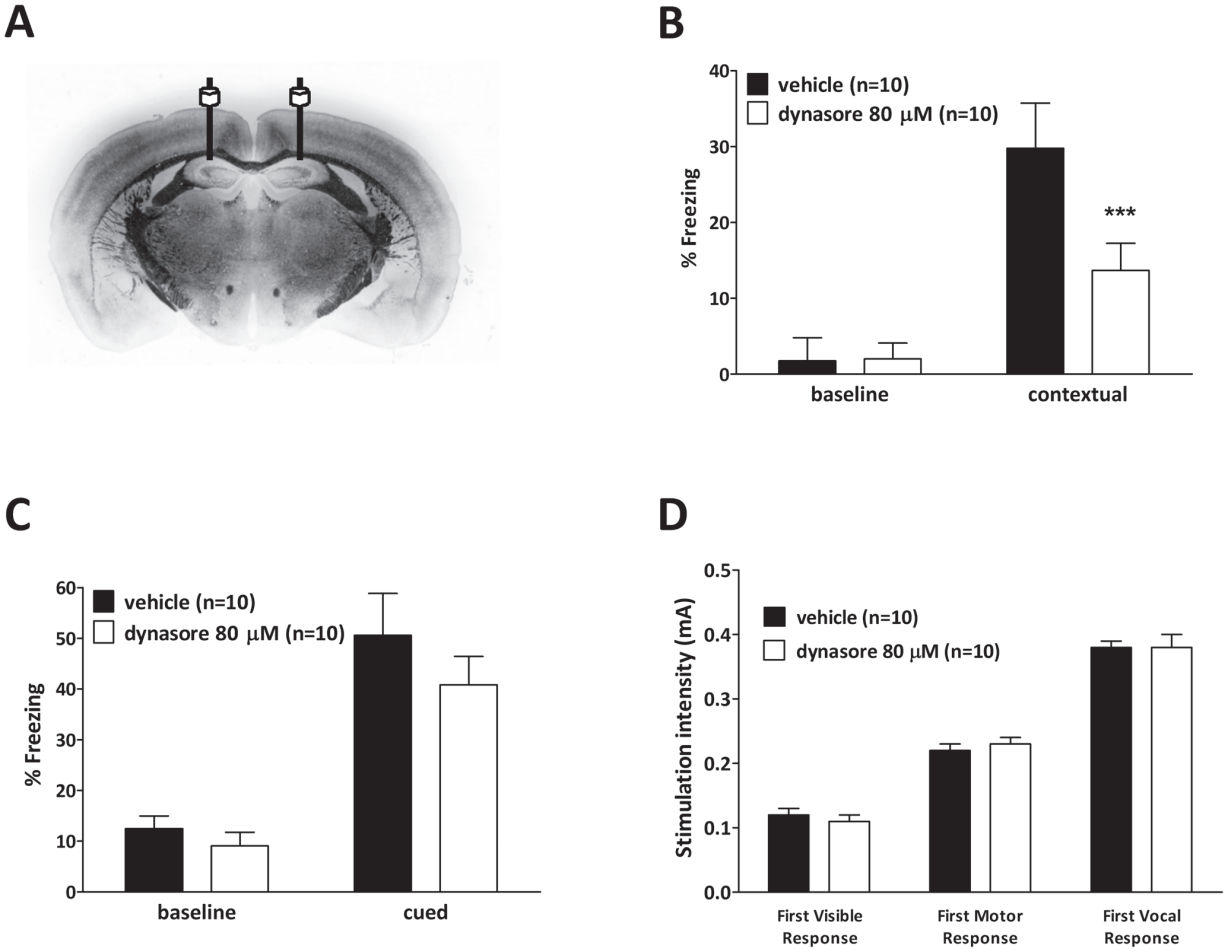
Dynamin inhibition by dynasore impairs associative memory. **A**, Schematic representation of the hippocampi bilaterally implanted with cannulas. **B**, Bilateral injections of dynasore (80 μM in a final volume of 1.5 μl over 1 minute) into dorsal hippocampi, 20 minutes before training, dramatically impairs contextual fear memory (open bars) compared to vehicle treated mice (black bars) (Mann-Whitney U_137, 73_ = 18.00, *p* = 0.00147). **C**, Mice do not show changes in cued fear conditioning following dynasore infusions (open bars) compared to vehicle-treated animals (black bars) (Mann-Whitney U_121, 89_ = 34.00, *p* = 0.2412). Each bar represents the average percent of time spent in freezing posture. Error bars indicate SEM. **D**, Sensory threshold was not affected regardless of treatment (n = 10).

In a first series of experiments, we checked whether memory is affected by dynasore, a synthetic non-peptidic inhibitor of dynamins [13]. The compound blocks both dynamin-dependent endocytosis [4] and dynamin-dependent evoked release [7] while it generates branched tubular membrane networks, capped by clathrin-coated pits upon intense exocytosis [14], similar to those observed in the neurons of dynamin 1-null mice [5]. We analyzed the effects of dynasore on contextual fear memory [15], a form of associative, hippocampal-dependent memory in which mice have to associate the environment they are exposed to with the occurrence of an aversive stimulus that is delivered during the training phase. Memory is then assessed via observation of freezing behavior upon re-exposure to the same context. Either dynasore or vehicle was administered via intrahippocampal bilateral injection to avoid any systemic side effect (Fig. 1A). Interestingly, dynasore infusion (80 μM, 20 minutes prior to the electric shock) reduced the amount of freezing compared to vehicle (Fig. 1B). By contrast, cued conditioning, a hippocampus-independent task [15] that was assessed 24 hours after contextual memory by exposing the animals to the tone in a novel environment, was not affected by dynasore (Fig. 1C). Similarly, assessment of the sensory threshold did not reveal any difference between dynasore- and vehicle-infused mice (Fig. 1D) suggesting that treatment with dynasore does not affect perception of the electric shock. Taken together, these data strongly support the possibility that dynamin inhibition impairs contextual fear memory.

### Dynamin is involved in synaptic fatigue evoked by high-frequency stimulation

**Figure 2:**
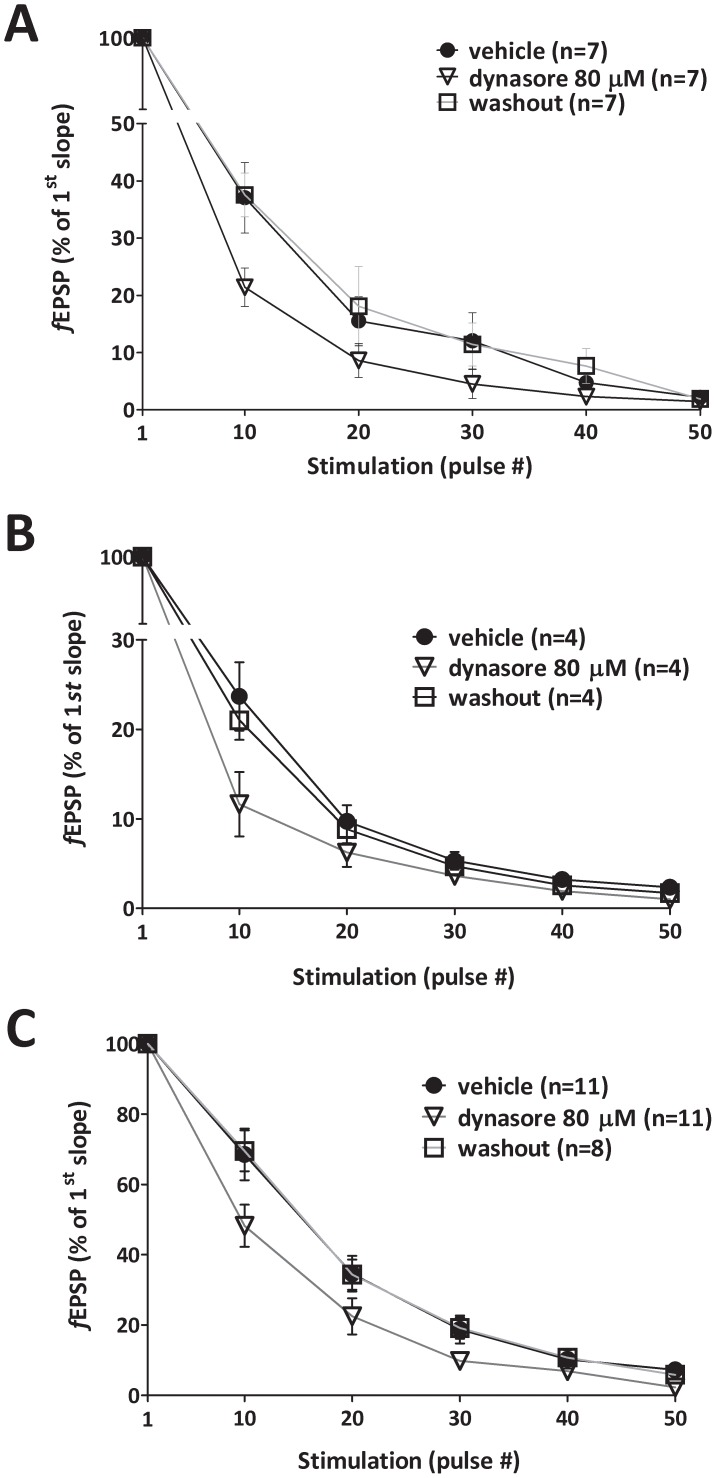
Dynamin inhibition by dynasore increases synaptic fatigue following sustained activity in hippocampus. **A**, Dynasore treatment (80 μM, open triangles) increases SF in slices that were previously treated with vehicle (black circles) (F_1,12_ = 6.395, *p* = 0.026). The increase is already present at the 10^th^ pulse during the stimulation (Mann-Whitney U_69, 36_ = 8.00, *p* = 0.0379). The effect was reversed by washout with vehicle (open squares). SF was induced by high frequency stimulation in the presence of D-APV (100 μM). **B**, SF was induced by high frequency stimulation (100 Hz, 1 second) in slices containing both D-APV (100 μM) and cyclothiazide (100 μM). Dynasore (80 μM, open triangles) further increases SF compared to fatigue of the same slices in the presence of vehicle (black circles) (F_1,12_ = 6.395, *p* = 0.026). SF increases in dynasore-treated slices already at the 10^th^ pulse during the tetanus (Mann-Whitney U_35, 10_ = 0.0001, *p* = 0.0159). The effect is reversed by washout with vehicle (open squares), re-establishing SF to the values obtained prior to dynasore perfusion. **C**, SF is induced by high frequency stimulation (100 Hz, 1 second) in vehicle-treated slices (black circles) containing both D-APV (100 μM), the GABA_A_ receptor blocker picrotoxin (30 μM), the GABA_B_ receptor blocker SCH 50911 (100 μM). Dynasore (80 μM, open triangles) further increases SF compared to fatigue of the same slices in the presence of vehicle (black circles) (F_1,12_ = 6.395, *p* = 0.026). SF increases in dynasore-treated slices already at the 10^th^ pulse during the tetanus (Mann-Whitney U_159, 94_ = 28.00, *p* = 0.0356). The effect is reversed by washout with vehicle (open squares), re-establishing SF to the values obtained prior to dynasore perfusion. Data shows dynasore-induced increase in SF is not associated to AMPA receptor desensitization or changes in GABA_A/B_ responsiveness. Error bars indicate SEM.

Memories are stored in the brain as short- and long-term changes in synaptic strength [16]. Because of this link, we next analyzed the effects of dynamin inhibition on synaptic functioning during high frequency stimulation, a type of sustained activity that is known to reinforce synapses. Prolonged, repetitive stimulation is known to cause synaptic fatigue (SF), a form of short-term plasticity generally reflecting a depletion of a readily releasable vesicle pool within the presynaptic terminal [17]. Perfusion of hippocampal slices with dynasore (80 μM for 20 minutes) accelerated SF evoked by a 100 Hz, 1 second, high frequency stimulation (Fig. 2A). These experiments were performed in the presence of the N-methyl-*d*-aspartate (NMDA) receptor blocker D-aminophosphovaleric acid (D-APV) (100 μM), leaving intact 2-amino-3-(5-methyl-3-oxo-1,2-oxazol-4-yl)-propanoic acid (AMPA) receptor-mediated events [17]. Perfusion with cyclothiazide (100 μM), to antagonize AMPA receptor desensitization and exclude the possibility that the cause of the heightened fatigue was a desensitization of postsynaptic receptors [18], [19], did not modify the decay rate produced by dynasore alone (Fig. 2B). Furthermore, perfusion with both picrotoxin (30 μM), a GABA**_A_** receptor antagonist, and SCH 50911 (100 μM), a GABA**_B_** receptor antagonist, applied to antagonize the GABAergic input, was not capable of protecting from the further decay rate induced by dynasore (Fig. 2C). Interestingly, the presence of the two antagonists slowed down the decay of the SF, as previously demonstrated [20]. Additionally, the effect of dynasore on SF in the presence of the two antagonists was weaker than in the other two conditions, probably due to augmentation in vesicle reuse produced by picrotoxin [20]. Taken together, these findings support the hypothesis that dynamin regulates release evoked by repetitive activity through presynaptic mechanisms.

### Dynamin inhibition reduces hippocampal long-term potentiation

**Figure 3:**
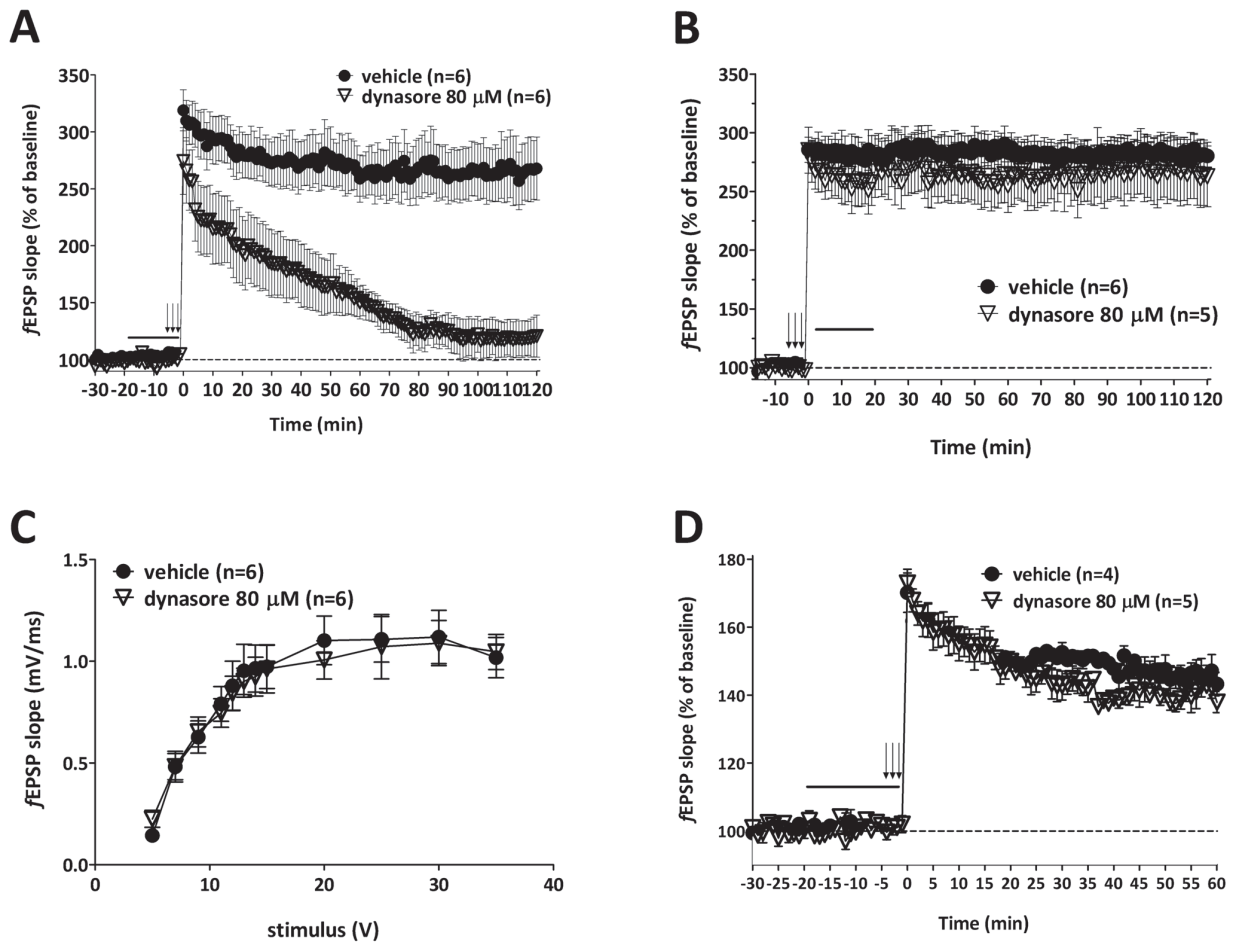
Dynamin inhibition by dynasore affects LTP, a type of synaptic plasticity due to sustained activity in hippocampus. **A**, Dynasore (80 μM, 20 minute perfusion, open triangles) decreases LTP induced by theta-burst stimulation in CA_3_–CA_1_ synapses compared to vehicle-treated slices (black circles)(F_1,10_ = 9.081, *p* = 0.013). The horizontal bar indicates the period of perfusion with dynasore before tetanic stimulation. **B**, Post-tetanus dynamin inhibition by dynasore (80 μm, 20 minute perfusion *after* the tetanus delivery, open triangle) induced by theta-burst stimulation in CA_3_–CA_1_ synapses compared to vehicle-treated slices (black circles; F_1,7_ = 0.209, *p* = 0.662). The horizontal bar indicates the period of perfusion with dynasore *after* tetanic stimulation. **C**, Basal synaptic transmission is unmodified by dynamin inhibition with dynasore. Averaged evoked field potential slopes as a function of stimulation intensity measured in volts (V) at CA_3_–CA_1_ synapses in slices do not show significant differences between vehicle-treated (black circles) and dynasore (80 μM, open triangles) treated slices (F_1,11_ = 40.081, *p* = 0.7013). **D**, Dynamin inhibition by dynasore (open triangles; 80 μm, 20 minute perfusion before the tetanus) does not produce changes in solely post-synaptic LTP induced by three tetani at 50 Hz for 1 second, each tetanus separated by 20 seconds, at the CA_3_–CA_1_ synapse compared to vehicle-treated slices (black circles; F1,8 = 1.538, p = 0.250). The horizontal bar indicates the period of perfusion with dynasore before tetanic stimulation. Error bars indicate SEM.

Since long-term potentiation (LTP) is an activity-dependent phenomenon widely proposed as a cellular model for learning and memory [21], we aimed to see whether mice display any LTP deficit in evoked hippocampal responses following dynamin inhibition. Dynasore perfusion (80 μM for 20 minutes) *before* (Fig. 3A) - but not after (Fig. 3B) a theta-burst stimulation (4 pulses at 100 Hz, with the bursts repeated at 5 Hz and each tetanus including 3 ten-burst trains separated by 15 seconds), markedly reduced CA_3_-CA_1_ LTP, suggesting a role of dynamin specifically related to the tetanus-mediated stimulation.

Changes in evoked responses during sustained activity were not associated, in turn, with significant modification of basal neurotransmission (Fig 3C). Treatment of mouse hippocampal slices with dynasore (80 μM for 20 minutes) did not affect *f*EPSP input/output relationships. These results are consistent with studies showing that basal evoked exocytosis is not affected by dynamin inhibition in rodent neurons [4], [5], [7]. Furthermore, they suggest that synaptic AMPA receptor currents in basal conditions are not affected by a brief dynamin block.

The theta-burst stimulation protocol used in the experiments reported above is known to produce a compound LTP involving both pre- and post-synaptic mechanisms [11]. As our data shown on Fig. 2 support the hypothesis that dynamin inhibition may regulate release evoked by repetitive activity through presynaptic mechanisms, we investigated whether dynamin inhibition may also reduce a form of LTP that does not require a strong presynaptic input for its induction (three tetani at 50 Hz, 1 sec each, 20 seconds interval between tetani) [11]. This different stimulation protocol was indeed able to produce a weaker LTP in vehicle-treated slices (Fig. 3D). However, no difference was observed in LTP between dynasore-treated (80 μM for 20 minutes) and vehicle-treated slices (Fig. 3D), indicating that dynasore yielded no apparent modification of LTP propensity at lower stimulation frequency.

### Dynamin inhibition reduces synaptic plasticity by impinging on pre-synaptic mechanisms evoked by high-frequency stimulation

**Figure 4:**
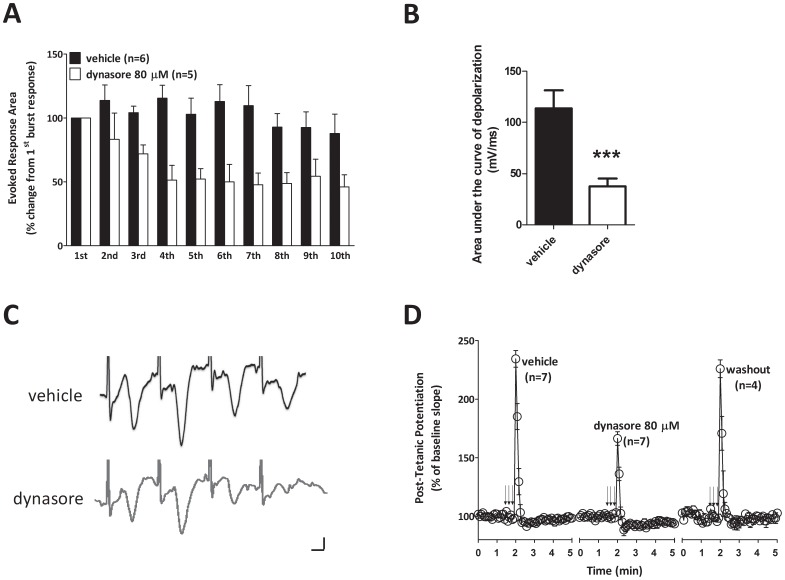
Dynamin inhibition affects presynaptic mechanisms underlying synaptic plasticity evoked by sustained activity. **A**, Tetanic efficiency, expressed as percent in evoked potential area across a train of 10 bursts at 5 Hz, each consisting of 4 pulses at 100 Hz, is reduced by dynasore (80 μM, open bars) with respect to percent response area in vehicle-treated slices (black bars) (F_1,9_ = 12.071, *p* = 0.007). The reduction reaches statistical significance at the third burst (Mann-Whitney U_51, 15_ = 0.00001, *p* = 0.0043); **B**, Dynasore (80 μM, open bars) already produces a partial reduction of the area within the first group of 4 pulses at 100 Hz compared to vehicle-treated slices (black bars); **C**, Raw *f*EPSP signals recorded during tetanic stimulation in vehicle-treated and dynasore (80 μM, 20 minutes before tetanus)-treated hippocampal slices. Calibration: 0.5 mV, 2 ms;. **D**, Perfusion of hippocampal slices with dynasore (80 μM) in the presence of D-APV (100 μM) for 20 minutes prior to theta-burst stimulation diminishes PTP to about 50% of the values obtained with vehicle perfusion (F_1,12_ = 6.924, *p* = 0.022). The effect was reversed by washout with vehicle. Error bars indicate SEM.

To test whether a possible decrease of neurotransmitter vesicle availability produced by dynamin inhibition would decrease tetanic stimulation efficacy in inducing plasticity, we measured the depolarization occurring *during* the tetanus [12]. We found a marked reduction in the area under the curve of depolarization after dynasore treatment (80 μM for 20 minutes) compared to vehicle-treated slices (Fig. 4A–B), suggesting a *reduction* of presynaptic input along the tetanus. Interestingly, the reduction in area was present within the first burst of 4 pulses at 100 Hz (Fig. 4C) and became statistically significant along the burst set with the third group (Mann-Whitney U_51, 15_ = 0.00001, *p* = 0.0043). This indicates that the efficacy of the theta-burst stimulation in triggering the compound LTP is not conserved upon dynamin inhibition, and supports the possibility that dynamin block reduces presynaptic input.

An additional evidence in favor of a dynamin inhibition-induced impairment of neurotransmitter released during sustained activity derived from studies on post-tetanic potentiation (PTP). This is a type of short-term synaptic plasticity dependent upon Ca^2+^ elevation within the presynaptic terminal during high frequency stimulation [17]. Theta-burst stimulation in slices treated with dynasore (80 μM for 20 minutes; in the presence of D-APV to block NMDA receptors, leaving intact AMPA receptor-mediated events) produced less PTP (Fig. 4D). Thus, similar to the SF and LTP experiments, the reduced potentiation upon dynasore treatment suggests a presynaptic deficit produced by dynamin inhibition during sustained activity.

### Dynamin inhibition does not affect paired-pulse facilitation evoked by a paired stimulation

**Figure 5:**
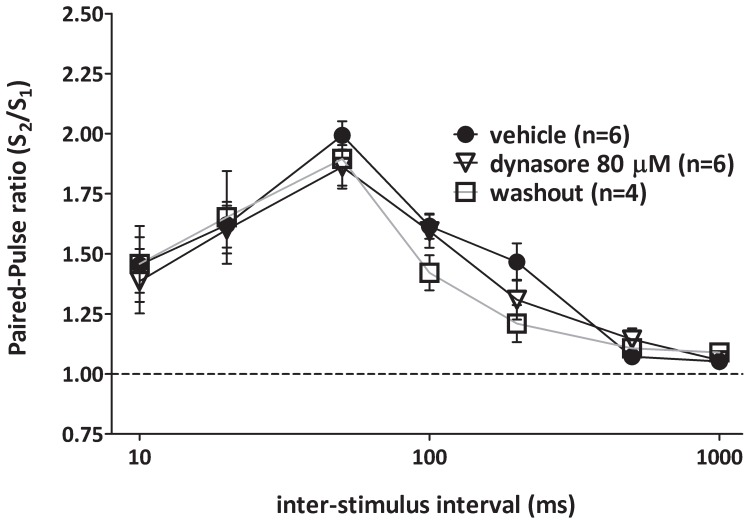
Dynamin inhibition with dynasore does not affect paired-pulse facilitation, a type of synaptic plasticity that is not linked with sustained activity. Paired-pulse-induced change is calculated as the ratio of the slope of the second evoked field potential to the first one at different interpulse intervals (1–1000 ms). Paired-pulse ratio is not modified in dynasore (80 μM, open triangles) treated slices with respect to vehicle-treated slices (black circles)(F1,12 = 0.339, p = 0.914). No effect is observed by subsequent washout with ACSF (open squares). Error bars indicate SEM.

Paired-pulse facilitation (PPF) is a short-term plasticity phenomenon that, differently than SF, PTP and LTP, is not due to sustained release. It occurs when a synaptic response is enhanced by a preceding stimulation of similar intensity, presumably elevating the Ca^2+^ concentration for a very short time [17]. We found that dynamin inhibition with dynasore (80 μM for 20 minutes) did not modify PPF (Fig. 5). This finding suggests that dynamin does not control short-term plasticity phenomena unless they are related to sustained/prolonged activity such as PTP or SF. The dissociation between effects of dynamin inhibition onto SF, PTP, LTP, and tetanus efficacy on one side and PPF on the other side is very interesting as it indicates that dynamin intervenes in different and mechanistically separated release phases. Such a phenomenon has already been described for SF, PPF and PTP which are known to have different intracellular mechanisms [22]. Indeed, KO animals for different types of synapsin, as well as other presynaptic proteins such as synaptogyrin I and synaptophysin, display a dissociation between different forms of short-term plasticity [23], [24].

### Synaptic plasticity and memory rely upon the dynamin 1 isoform

**Figure 6:**
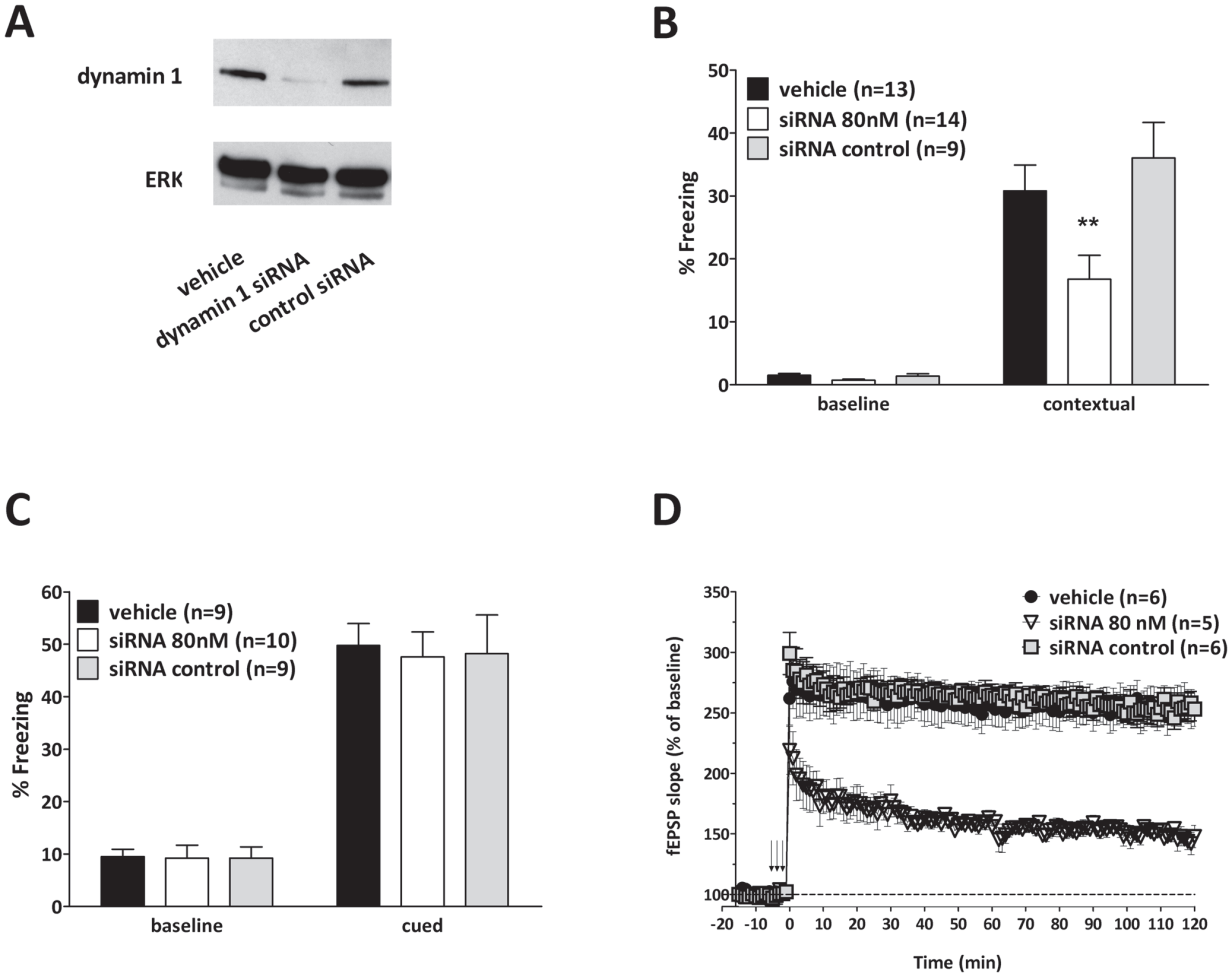
Selective dynamin 1 inhibition through siRNA impairs both synaptic plasticity and associative memory. **A**, siRNA specific for murine dynamin 1 reduces protein expression. An example of western blot showing that Penetratin 1- conjugated dynamin 1 siRNA reduces protein expression. Cells are lysed 48 hours after the treatment with siRNA. Dynamin 1 is detected using a rabbit polyclonal anti-dynamin1 antibody. Penetratin 1- conjugated Control siRNA, that does not affect dynamin 1 expression, does not change protein levels. n = 3 for each group. **B**, Penetratin 1- conjugated dynamin 1 siRNA (open bars) (80 nM in a final volume of 1.5 μl over 1 minute, bilateral injections twice a day for 3 days) impairs contextual fear memory compared to control siRNA infused mice (grey bars) (Mann-Whitney U_150, 126_ = 21.00, *p* = 0.0089). Moreover, control siRNA infused mice show similar amount of freezing as vehicle-infused animals (black bars). **C**, Penetratin 1- conjugated dynamin 1 siRNA (open bars) does not modify cued fear memory compared to Penetratin 1- conjugated Control siRNA (grey bars) (Mann-Whitney U_99, 90_ = 44.50, *p* = 1.00) in mice previously tested for contextual fear conditioning at 24 hours after the shock. **D**, Bilateral infusions of Penetratin 1- conjugated dynamin 1 siRNA (open triangles) (80 nM in a final volume of 1.5 μl over 1 minute, repeated 2 times a day for three days) into dorsal hippocampi decrease LTP compared to Penetratin 1- conjugated Control siRNA treatment (grey squares) (F_1,9_ = 5.578, *p* = 0.001). As an internal control, slices from vehicle-infused animals (black circles) show similar amounts of potentiation as those from Penetratin 1- conjugated Control siRNA treated animals. Error bars indicate SEM.

Dynamin has three different isoforms, dynamin 1–3. Given that dynasore is not selective toward a specific dynamin isoform [13] and to address concerns related to the specificity of pharmacological tools [7], we next examined both synaptic plasticity and associative memory upon knocking down dynamin. Dynamin 1 is pre-synaptically located and highly-expressed in neurons, whereas dynamin 2 is located both pre- and post-synaptically and dynamin 3 is localized mostly post-synaptically [25]. Therefore, we decided to block dynamin 1. Dynamin 1 knockouts are lethal at 2 weeks of age [5]. Thus, we used a different genetic approach in which we conjugated a small interfering RNA (siRNA) against dynamin 1 with penetratin [26]. In preliminary experiments, we demonstrated by quantitative Western blot analysis that dynamin 1 was reduced by approximately 90% in primary hippocampal cultures that were exposed to Penetratin1-conjugated dynamin 1-siRNA (80 nM) for 48 hours prior to cell collection (Fig. 6A), whereas a control siRNA did not affect dynamin 1 expression. Next, we infused Penetratin1-conjugated dynamin 1-siRNA (80 nM in a final volume of 1.5 μl over 1 minute, bilateral intrahippocampal infusions twice a day for 3 days prior to electrophysiology or behavior). We found a reduction in both contextual fear memory and LTP (Fig. 6B–D). By contrast, controls, treated with intrahippocampal infusion of Penetratin 1-conjugated siRNA against luciferase, did not reveal any effect on memory and LTP. These findings suggest a prominent role for dynamin 1 in associative fear memory and synaptic plasticity phenomena associated with sustained activity.

## Discussion

The main finding of the present work is that dynamin 1 is important for the formation of associative memory in hippocampus. An additional finding is that both short- and long-term plasticity phenomena caused by fast and prolonged activity and thought to be linked to memory formation, depend upon dynamin function. Specifically, we have demonstrated that SF, LTP, PTP, and tetanus efficacy, all phenomena due to sustained release, are markedly reduced following dynamin inhibition.

The involvement of dynamin 1 in memory function opens new perspectives to studies on the function of the protein. Specifically, it translates effects of inhibition of this isoform into animal behavior, and memory in particular. These data are consistent with the observation that *shibire^ts1^*, a temperature-sensitive dynamin mutant gene in *Drosophila melanogaster*, alters neuronal mechanisms that are thought to be linked to learning and memory [27]. Similar to dynamin, two other synaptic proteins, the t-SNARE proteins SNAP-25 and syntaxin-1, are also required for memory formation [28], [29]. Interestingly, long-term memory formation in fresh water pond snail *Lymnaea stagnalis* is associated with increased expression of syntaxin 1 and dynamin 1 coincident with elevated CREB 1 levels, and knockdown of CREB1 prevented memory consolidation and reduced expression of both syntaxin and dynamin 1 [30]. Moreover, hippocampal dynamin-binding protein mRNA expression is reduced with aging in rats undergoing an inhibitory avoidance task [31]. Finally, a relationship between dynamin 1 and memory was hypothesized in a manuscript in which *Btbd9* knock-out mice displayed an increase in dynamin 1 expression together with enhanced synaptic plasticity and memory [32]. Taken together, these observations support the hypothesis that dynamin is activated during memory processes concurrently with other proteins acting in the presynaptic terminal.

The role of different forms of plasticity involving dynamin in evoked release during sustained activity is also very interesting. It is noteworthy to remark the fact that memory formation is not always linearly paired with the level of neurotransmitter release as one might assume *a priori*, given that other proteins are still able to increase memory and synaptic plasticity while decreasing the presynaptic input [33], [34]. In the case of dynamin inhibition, instead, we did observe a parallelism between memory and plasticity and availability of presynaptic input. Consistent with this finding, it has been shown that dynamin binds to the vesicle-associated presynaptic protein synaptophysin, such that disruption of the interaction between the two proteins results in a decrease of transmitter release during high-frequency stimulation [35].

LTP at CA_3_–CA_1_ connection is not a single-compartment phenomenon, but rather a mix of both presynaptic and postsynaptic processes that independently strengthen the synapse. Theta-burst CA_3_-CA_1_ LTP activates second messanger cascades including the cAMP-PKA pathway either in the presynaptic terminal to directly boost presynaptic function or in the postsynaptic site to produce a retrograde messenger as well as activate glutamate receptors and L-voltage-gated calcium channels in the post-synaptic neuron [36]–[40]. By contrast, LTP induced with a weaker tetanic stimulation (three tetani at 50 Hz, 1 sec each, 20 second interval between tetani) is not affected by either PKA or L-voltage gated calcium channels inhibition. Moreover, this form of LTP induced with a weaker tetanic stimulation is not accompanied by changes in signal decay rate of markers used to monitor presynaptic functions (either FM 1–43 staining or spH fluorescence) [11], [41], indicating that it is not sensitive to presynaptic changes. In our studies, we found that LTP induced through a weak tetanic stimulation was not affected by the dynasore treatment. This finding together with the lack of modification in basal evoked post-synaptic responses indicates that a brief application of dynasore does not interfere with postsynaptic mechanisms. Thus, it is likely that dynamin regulates LTP induced at high (theta-burst at 100 Hz) rather than at low frequency (50 Hz) stimulation. Moreover, these data further support the idea that dynamin functions in plasticity phenomena consist of modulating vesicle release efficiency.

Recent evaluations of vesicle dynamics [7], [42], [43] might provide an explanation for the memory and plasticity impairment following dynamin inhibition. When dynamin is unable to contribute to the endocytotic processes, whether by affecting the vesicle pools or by disrupting the release machinery activity, decreased vesicle availability may affect the neuron capability for an effective synaptic release sustaining memory phenomena. This effect might be particularly evident when assessing synaptic plasticity during the high frequency tetanic stimulation, as highlighted by the reduced evoked potential during tetanus in the presence of dynamin blockage and by the lack of any effect of dynasore when administered *after* the tetanic stimulation suggesting, in turn, that dynamin inhibition affects memory acquisition. Dynamin activity becomes essential for memory and plasticity induced by high frequency stimulations, such as theta burst stimulation, in line with the independent findings of Ferguson et al [5] and Bartolomé-Martín et al [14] showing that dynamin inhibition generates branched tubular membrane networks or intense clusterization of vesicles, capped by clathrin-coated pits upon intense exocytosis, limiting *de facto* the vesicle availability for prompt release. This suggests that, at least at high frequency stimulation, even the slow vesicle recovery due to the classic clathrin-dependent/dynamin-dependent endocytosis is necessary to compensate the intense vesicle use for rapid release due to tetanic stimulation. Our data on contextual fear conditioning and LTP are novel because they correlate the possible efficiency of dynamin-dependent vesicle availability selectively with memory and plasticity phenomena, while our data on basal synaptic transmission confirm that synaptic potentials not evoked by sustained release are not affected by residual vesicle availability upon dynamin inhibition. Further investigations, beyond the scope of this work, are required to discern whether solely the classical or any alternative endocytotic pathways or even non endocytosis-related mechanisms are critical for dynamin-dependent memory and synaptic plasticity.

In our studies, we provide both pharmacologic and genetic proof of the involvement of dynamin in memory formation. There are several advantages with using a genetic approach in addition to a pharmacologic one. The experiments with dynamin 1 siRNA provide an independent genetic validation of the findings with dynasore. Furthermore, they demonstrate that the isoform 1 of dynamin is active in memory and synaptic plasticity. Finally, they offer precious insight into the complementarity of the three major dynamin isoforms. Neither dynamin 2 nor dynamin 3 were capable of compensating for the detrimental effect exerted on cognition and plasticity by selective dynamin 1 knockdown obtained through siRNA technique. Similarly, the presence of dynamin 2 and 3 was not able to avoid death in dynamin 1-knockout mice at two weeks of age [5]. Interestingly, dynamin 2 is involved in slower forms of endocytosis than dynamin 1 [44]. By contrast, dynamin 1, whose over-expression triggers recruitment of clathrin–independent and dynamin-dependent vesicles, regulates both fast and slow endocytosis [45]. Therefore, the differential ability of the dynamin isoforms in modulating the neurotransmitter vesicle availability over time may be relevant in terms of release during fast stimulation [46] and controlling plasticity underlying the onset of associative memory.

Genetic ablation of dynamin 1 produced a less marked effect onto synaptic plasticity than the pharmacologic block with dynasore. Possible explanations for the incomplete block of LTP by the siRNA are likely to be linked to the fact that dynasore inhibits multiple dynamin isoforms whereas the siRNA blocks selectively dynamin 1, and/or that dynasore affects substrates other than dynamins [47]. Interestingly, astrocytic dynamin 2 controls the recycling of the tissue plasminogen activator, a factor regulating NMDA functions and glutamate release and regulated itself by ambient glutamate [48]. Along this direction, dynamin 2 in glia might cooperate with dynamins in neurons to modulate synaptic plasticity. Additionally, dynamin 3 but not dynamin 2, when overexpressed, partially rescues the dynamin 1 knock-out phenotype. Consistent with this observation, dynamin 1 and 3 conserve phosphorylation sites on key residues that are controlling protein-protein interactions. Instead, phospho-sites on dynamin 2 are important for other functions [48]. Nevertheless, one should keep in mind that dynamin 1 is the major dynamin isoform in neurons [5] and levels of dynamin 2 and 3, as well as many other proteins involved in synaptic transmission and endocytosis, are not changed in dynamin 1 knock-out mice [49].

As previously reported, the level of neurotransmitter release during ongoing activity may be regulated by the availability of release-ready vesicles and, therefore, depends on the speed of vesicle recruitment. In that sense, synaptic vesicles need to undergo fast recycling to prevent depletion of the synaptic vesicle pool [50]. Interfering with the endocytosis produces a fast, stimulation-frequency-dependent depression of exocytosis [6]. This would prompt the likely idea that lack of release-prone vesicles is actually due to the absence of recycled vesicles following consumption of synaptic vesicles through exocytosis and slowed rates of vesicle recycling producing, in turn, a reduction of synaptic vesicles in the readily releasable pool [4], [6]. However, this idea has been confuted several times. For instance, both rapid and slow endocytosis do not recycle vesicles within the readily releasable pool [42]. Moreover, the exocytosis and endocytosis are thought to be functionally independent of each other and therefore the endocytosis block does not immediately affect subsequent exocytosis [4], [6]. Furthermore, the inhibition of vesicle replenishment by folimycin (limiting *de facto* the number of vesicle recovered and ready to be reused) does not mimic the clear stimulation-frequency-dependent release depression after treatment with dynasore or a clathrin inhibitor treatment, while it efficiently blocks vesicular refilling [50]. Instead, these results suggest that dynasore would interfere with the process of rapid clearance of exocytosed vesicle components from the synaptic release sites [50], as previously postulated [6], [51]. These components include synaptotagmin 1, a calcium sensor protein involved in both exo- and endocytosis [52]–[54], and calmodulin [55]. If this interpretation of dynasore effect is conceivable, this means that dynamin, along with other endocytic proteins, is essential for sustained synaptic transmission well beyond its well-established role in endocytosis [50].
